# A Gain-of-Function Mutation in TRPA1 Causes Familial Episodic Pain Syndrome

**DOI:** 10.1016/j.neuron.2010.04.030

**Published:** 2010-06-10

**Authors:** Barbara Kremeyer, Francisco Lopera, James J. Cox, Aliakmal Momin, Francois Rugiero, Steve Marsh, C. Geoffrey Woods, Nicholas G. Jones, Kathryn J. Paterson, Florence R. Fricker, Andrés Villegas, Natalia Acosta, Nicolás G. Pineda-Trujillo, Juan Diego Ramírez, Julián Zea, Mari-Wyn Burley, Gabriel Bedoya, David L.H. Bennett, John N. Wood, Andrés Ruiz-Linares

**Affiliations:** 1 Department of Genetics, Evolution and Environment, University College London, London WC1E 6BT, UK; 2 Molecular Nociception Group, Wolfson Institute for Biomedical Research, University College London, London WC1E 6BT, UK; 3 Department of Neuroscience, Physiology and Pharmacology, University College London, London WC1E 6BT, UK; 4 Grupo de Neurociencias, Facultad de Medicina, Universidad de Antioquia, Medellín, Colombia; 5 Grupo de Mapeo Genético, Facultad de Medicina, Universidad de Antioquia, Medellín, Colombia; 6 Department of Medical Genetics, Cambridge Institute for Medical Research, Addenbrooke's Hospital, Cambridge CB2 0XY, UK; 7 Department of Neurorestoration, Wolfson CARD, Hodgkin Building, Guy's Campus, King's College London, London SE1 1UL, UK; 8 World Class University Department of Molecular Medicine and Biopharmaceutical Sciences, Seoul National University, Korea

**Keywords:** HUMDISEASE, MOLNEURO, SIGNALING

## Abstract

Human monogenic pain syndromes have provided important insights into the molecular mechanisms that underlie normal and pathological pain states. We describe an autosomal-dominant familial episodic pain syndrome characterized by episodes of debilitating upper body pain, triggered by fasting and physical stress. Linkage and haplotype analysis mapped this phenotype to a 25 cM region on chromosome 8q12–8q13. Candidate gene sequencing identified a point mutation (N855S) in the S4 transmembrane segment of TRPA1, a key sensor for environmental irritants. The mutant channel showed a normal pharmacological profile but altered biophysical properties, with a 5-fold increase in inward current on activation at normal resting potentials. Quantitative sensory testing demonstrated normal baseline sensory thresholds but an enhanced secondary hyperalgesia to punctate stimuli on treatment with mustard oil. TRPA1 antagonists inhibit the mutant channel, promising a useful therapy for this disorder. Our findings provide evidence that variation in the *TRPA1* gene can alter pain perception in humans.

**Video Abstract:**

## Highlights

► We describe a novel human autosomal-dominant pain syndrome (FEPS) ► We demonstrate linkage of FEPS to a TRPA1 N855S point mutation ► FEPS patients show debilitating pain on fasting and physical stress ► N855S TRPA1 channels show enhanced inward currents but unaltered pharmacology

## Introduction

Inherited human neurological disorders caused by mutations in ion channels encompass a diverse range of conditions including pathological pain states (Indo, 2001, Yang et al., 2004, Einarsdottir et al., 2004, Cox et al., 2006, Fertleman et al., 2006, Foulkes and Wood, 2008). Voltage-gated Na^+^ and Ca^2+^ channelopathies account for many cases of familial hemiplegic migraine and epilepsy, while K^+^ channel mutations have been associated with epilepsy, peripheral nerve hyperexcitability, and ataxia (Kullmann and Hanna, 2002). Dominant gain-of-function mutations of the peripheral nervous system sodium channel Na_V_1.7 have been linked to the painful disorders paroxysmal extreme pain disorder and erythermalgia (Yang et al., 2004, Fertleman et al., 2006, Dib-Hajj et al., 2007). Loss of functional Na_V_1.7 channel activity leads to congenital insensitivity to pain (Cox et al., 2006).

Transient receptor potential (TRP) channels are cation channels that are implicated in all aspects of sensation, including vision, olfaction, mechanosensation, thermosensation, and damage sensing (Nilius, 2007). However, no human heritable disorders of pain sensation have as yet been linked to mutations in TRP channels, despite knockout mouse studies that suggest a number of TRPs play an important role in pain pathways (Cregg et al., 2010). Mutations in TRPV4 (which is activated by innocuous heat and hypotonicity and is a putative mechanosensor) do not alter pain responses, but underlie two different neurodegenerative diseases, scapuloperoneal spinal muscular atrophy and Charcot-Marie-Tooth disease type 2C (Auer-Grumbach et al., 2010, Deng et al., 2010, Landoure et al., 2010) as well as two forms of skeletal dysplasia (Rock et al., 2008, Krakow et al., 2009). TRPA1 is expressed in primary afferent nociceptors in rodents and man (Story et al., 2003, Anand et al., 2008) and has been shown to play an important role in the response to environmental irritants in mouse models (Kwan et al., 2006, Bautista et al., 2006, McMahon and Wood, 2006). There is also strong evidence that TRPA1 is gated by cold temperatures and debate about whether the channel is mechanosensitive (Kwan et al., 2006, Rugiero and Wood, 2009). Here we describe the identification of a point mutation in the S4 domain of TRPA1 that underlies an autosomal dominant Mendelian heritable episodic pain syndrome identified in a family from Colombia, South America. Episodes are triggered by conditions of fatigue, fasting, and cold and result in severe pain localized principally to the upper body. By comparing the biophysical properties of the expressed mutant channel with wild-type (WT) TRPA1, we have been able to identify a mechanism that is likely to underlie the painful episodes. To our knowledge, this is the first example of a human pain-associated TRP channelopathy and is likely to be followed by further genetic insights into the role of TRP channels in pain pathways as pain-related genome-wide association studies come to fruition.

## Results

### Mapping the Familial Episodic Pain Syndrome Mutation

Individuals with FEPS present with episodes of debilitating upper body pain starting in infancy that are usually triggered by fasting or fatigue (illness, cold temperature, and physical exertion being contributory factors). Triggers did not start attacks with 100% reliability and often a combination of factors are needed; a typical example would be swimming in cold water not having eaten for a number of hours. These episodes have a typical duration of ∼1.5 hr, starting with a prodromal phase (which can sometimes be aborted, mainly by resting and eating), followed by intense pain, and terminating in a period of exhaustion and deep sleep.

**Figure 1:**
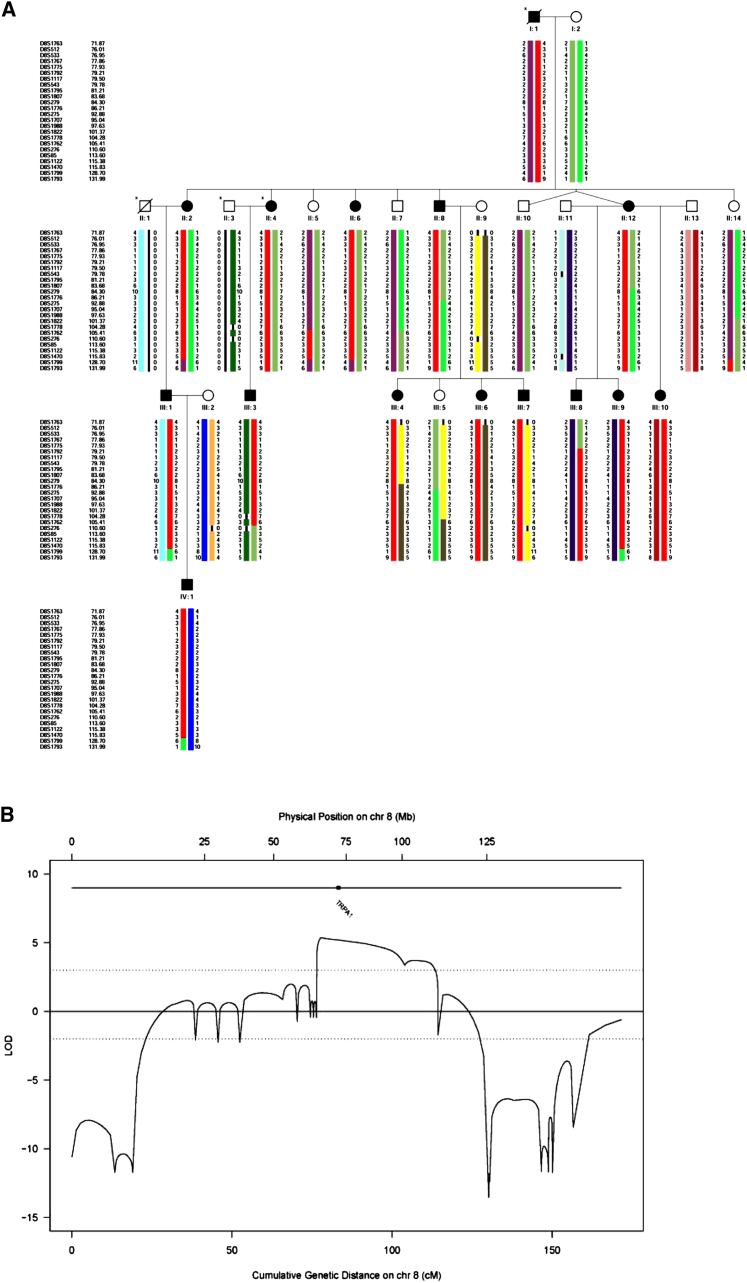
Genetic Mapping of the Mutant Locus Underlying FEPS (A) Pedigree segregating FEPS. Microsatellite haplotypes spanning the linkage peak on chromosome 8q12.1–8q24.1 identified in the whole-genome scan. The haplotypes shown were reconstructed using markers from the whole-genome scan and fine-mapping stages. The haplotype cosegregating with the pain phenotype is shown in red. The minimal critical region spans 25 cM and is flanked by marker D8S1775 on the centromeric end and marker D8S1762 on the telomeric end (informative recombinants individuals being III:8 and II:5, respectively). Only individuals included in the linkage analysis are shown (individuals with an asterisk were unavailable for genotyping). (B) Multipoint LOD scores obtained on chromosome 8 (including fine-mapping markers) and location of the *TRPA1* gene. Physical distance (in Mb) is shown at the top and genetic distance (in cM) at the bottom. The dotted lines indicate the LOD score thresholds of 3 and −2 (i.e., significant evidence for or against linkage, respectively).

The period of intense pain is accompanied by breathing difficulties, tachycardia, sweating, generalized pallor, peribuccal cyanosis, and stiffness of the abdominal wall. Affected individuals reported no altered pain sensitivity outside the episodes. They had a normal neurological examination, including normal sensitivity to light touch, pin prick, vibration sense, and joint position sense. Other than the Colombian pedigree described here we are not aware of any previous reports of this clinical syndrome (see Supplemental Information for illustrative case history and Tables S1–S4 summarizing clinical features, available online). A total of 21 affected family members in four generations were identified by pedigree extension, consistent with an autosomal dominant mode of inheritance (individuals included in the study are shown in Figure 1A).

**Figure 2:**
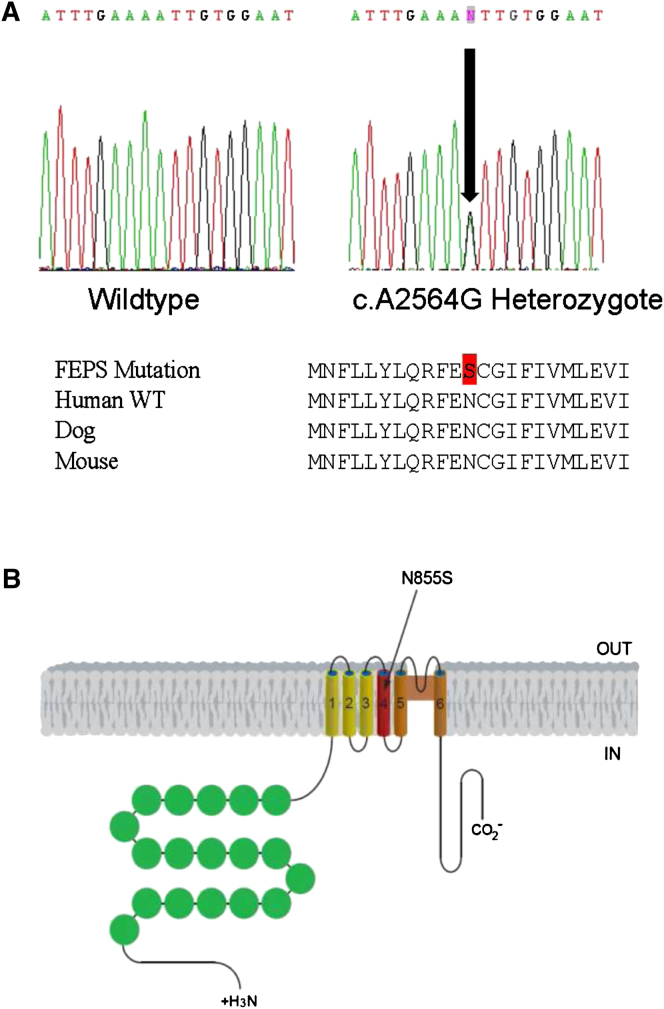
Identification of a Point Mutation Underlying FEPS (A) Sequence chromatogram showing the *TRPA1* mutation identified in the FEPS family. The arrow indicates the location of the mutation. Below is a selection of mammalian *TRPA1* sequences showing that the mutation site region is evolutionarily conserved. (B) Schematic representation of the TRPA1 channel. The substitution (S) identified in the FEPS family occurs in asparagine (N) 855 located in putative transmembrane segment S4.

We performed a genome-wide linkage scan with ∼550 microsatellite markers in 13 affected and 10 unaffected family members (Figure 1A). Parametric linkage analysis produced positive LOD scores across chromosome 8q12.1–8q24.1, with a maximum two-point LOD score of 4.18 for marker D8S512 (at θ = 0) and a multipoint LOD score of 4.42 between markers D8S512 and D8S279 (at 8q12.3–8q13.3). Typing of additional microsatellite markers in the region resulted in a maximum multipoint LOD score of 5.36 at position 79 cM on chromosome 8q13 and haplotype analysis further narrowed down the candidate region to an interval of ∼25 cM spanning chromosome 8q13.2–8q22.2 (Figure 1). Candidate gene sequencing in affected individuals identified an A to G transition in exon 22, at position 2564 of the TRP channel member *TRPA1* cDNA (c.A2564G; Figure 2A). This change was observed in all affected individuals but not in unaffected family members. Sequencing of 139 ethnically matched unaffected controls failed to detect the c.A2564G mutation in the general population. This mutation results in the substitution of an asparagine by a serine (N855S) in the putative transmembrane segment S4 of TRPA1 (Figure 2B). TRPA1, which contains N-terminal ankyrin repeats, is a homolog of the NOMPC channel involved in hearing in *Drosophila*. However, knockout mouse studies have demonstrated an important role for TRPA1 in response to irritant chemicals, but not hearing (Kwan et al., 2006, Bautista et al., 2006). Zebrafish TRPA1 is also mechanically insensitive (Prober et al., 2008).

### Psychophysical Studies of FEPS Patients

**Figure 3:**
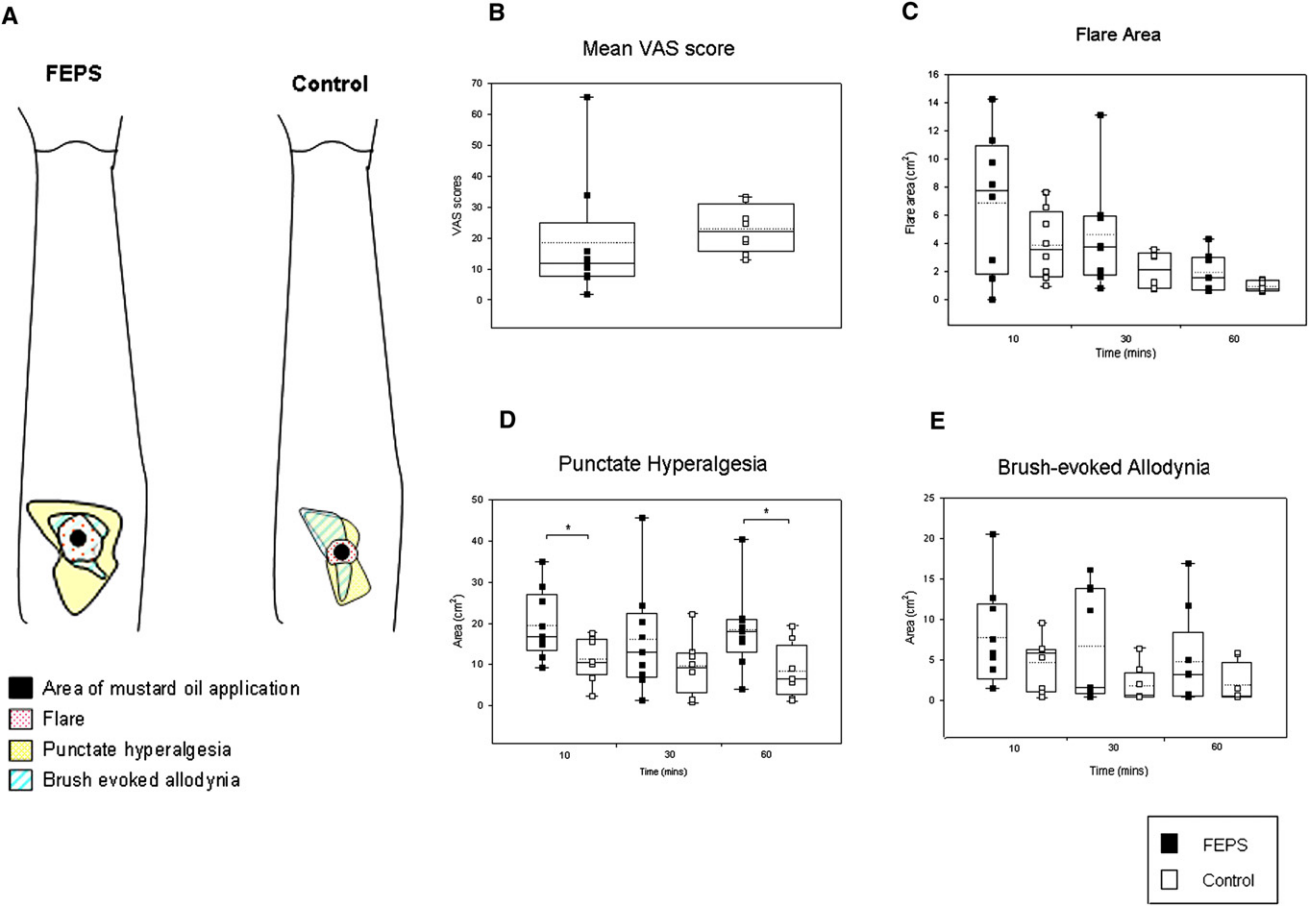
Increased Area of Punctate Hyperalgesia in Patients with FEPS after Topical Mustard Oil Application (A) The area of flare, secondary hyperalgesia to punctate stimuli, and brush-evoked allodynia 10 min after mustard oil application in a patient with FEPS and a family control. (B–E) Comparison of pain assessed by VAS during topical mustard oil application (B) and the area of flare (C), punctate hyperalgesia (D), and brush-evoked allodynia (E) at 10, 30, and 60 min after application in FEPS patients versus family controls. All assessments were performed in the same nine cases and eight family controls, except in (C) where only eight cases where examined. The boxes span the 25th to 75th percentile with the median represented as a solid line and the mean as a dotted line, the whiskers show the full range of the data. ^∗^p < 0.05, unpaired t test.

Skin biopsies were obtained from three subjects with the N855S TRPA1 mutation and three unaffected relatives. Both the morphology and density of intraepidermal nerve fibers (revealed by immunostaining with the pan-neuronal marker PGP 9.5) were normal (Figure S1). Quantitative sensory testing (QST) was performed in nine individuals with FEPS and in eight unaffected relatives. No significant difference was observed in tactile detection threshold, vibration detection threshold, or cold, heat, or pressure pain detection threshold in mutation carriers (Table S4). Mustard oil (Allyl isothiocyanate) is known to activate TRPA1 and in humans it has been shown to produce ongoing pain, a cutaneous flare response, and sensitization of the nociceptive system (Koltzenburg et al., 1992, Jordt et al., 2004). No significant difference was observed in the pain response [as assessed by visual analog scale (VAS)] during application of 50% mustard oil when comparing mutation carriers and non-carriers (Figure 3B). There was a (non-significant) increase in the mean flare area comparing FEPS patients versus control (SD in parentheses): 7.2 cm^2^ (±4.6) and 3.9 (±2.4), respectively (p = 0.1 unpaired t test; Figure 3C). Some (4/8) FEPS patients developed very large flares (of over 8 cm^2^) at 10 min after mustard oil application, whereas this reaction was not seen in the controls. Mutation carriers also showed a significant increase in the area of punctate hyperalgesia at 10 and 60 min after mustard oil application (p < 0.05, unpaired t test; Figure 3D) and a (non-significant) increase in the area of brush-evoked allodynia (Figure 3E). We were not able to perform extensive dose-response studies using mustard oil, however, as an initial trial (to assess tolerability) of 0.5% mustard oil was applied to the volar forearm and this did not evoke a response in either FEPS patients or control.

### Biophysical Studies of the FEPS-Associated TRPA1 Mutant Channel

**Figure 4:**
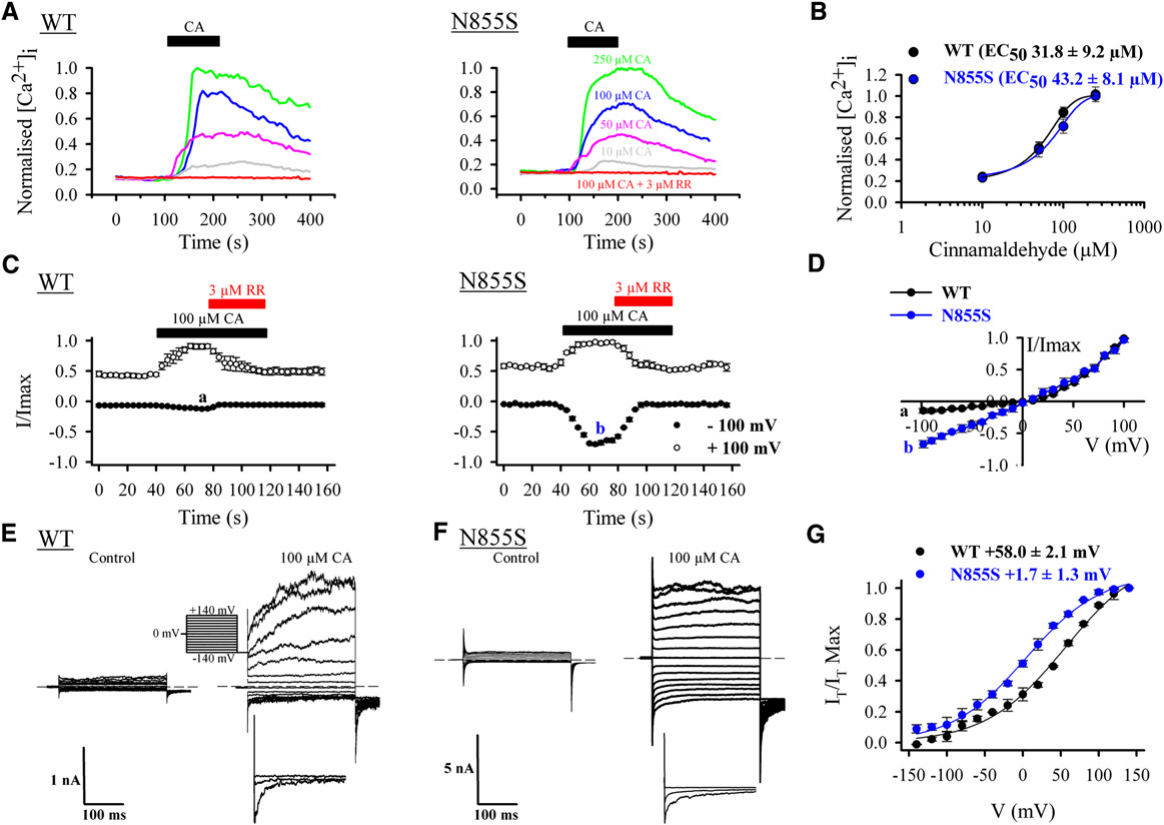
Pharmacological and Biophysical Analysis of hTRPA1-WT and hTRPA1-N855S (A) Intracellular calcium response to 250 μM, 100 μM, 50 μM, and 10 μM CA and to 100 μM CA in the presence of 3 μM ruthenium red (RR). Results for HEK293 cells expressing hTRPA1-WT are shown on the left and for hTRPA1-N855S on the right. Horizontal bars at the top indicate the time of CA application. (B) Dose-response curve of CA-evoked calcium responses for hTRPA1-WT and hTRPA1-N855S. [Ca^2+^]_I_ normalized to maximum calcium response to 250 μM CA. Traces represent average [Ca^2+^]_I_ from 20–30 cells. Data were fit to the Hill equation. (C) HEK293 cells expressing hTRPA1-WT (left; n = 5) or hTRPA1-N855S (right; n = 6) show activation by CA (100 μM) and inhibition by ruthenium red (RR). Currents were recorded at +100 mV and –100 mV and are normalized to current at +100 mV. Letters denote time point at which voltage ramps (shown in D) were acquired to generate current-voltage relationships. (D) Average current-voltage relationship of hTRPA1-WT and hTRPA1-N855S in the presence of 100 μM CA. Currents are normalized to +100 mV. (E) Whole-cell current traces of HEK293 cells expressing hTRPA1-WT in response to the indicated voltage step protocol in the absence (left) and presence (right) of 100 μM CA. Bottom panel shows higher resolution of normalized tail current in response to a step to −140, 0, and +140 mV. Dotted line shows zero current level. (F) Same as (E) but in HEK293 cells expressing hTRPA1-N855S. (G) Mean steady-state activation curves obtained from tail currents (I_T_) at −140 mV for hTRPA1-WT (n = 5) and hTRPA1-N855S (n = 6) in the presence of CA. The midpoints of voltage activation (V1/2) for the WT and mutant channels are indicated at the top. Error bars in all plots represent SEM across individual cell measurements.

Clones encoding the WT and N855S mutant human TRPA1 channel were expressed in HEK293 cells and characterized electrophysiologically using the whole cell patch configuration (Story et al., 2003). Calcium imaging studies used FURA-2-loaded HEK293 cells and OpenLab software as described previously (Abrahamsen et al., 2008). The half maximum effective concentration (EC_50_) for cinnamaldehyde (CA), a ligand that covalently modifies and activates TRPA1, was similar in both WT and mutant channels and in the range of the reported value for TRPA1—61 μM (Bandell et al., 2004) (Figures 4A and 4B). This is consistent with the mutation being distant from the N-terminal region, which has been implicated in CA binding (Hinman et al., 2006, Macpherson et al., 2007). However, the current-voltage relationship of the mutant channel revealed by ramp protocols was dramatically altered by the N855S mutation. Current-voltage plots in transfected cells in the absence of agonist were identical with both WT and mutant channels (Figure S2A). On agonist activation, inward current at normal neuronal resting potentials (−60 to −70 mV) showed that the mutant channel carried considerably more current than the outwardly rectifying WT channel (Figures 4C and 4D). While outward currents were identical at +100 mV in WT and mutant channels, at −100 mV the mutant channel carried 5.4-fold more current (Figure 4C, WT: −100 mV = −0.13 ± 0.02, +100 mV = 0.93 ± 0.04; mutant: −100 mV = −0.68 ± 0.04, +100 mV = −0.95 ± 0.03). At normal neuronal resting potentials, >4-fold increase in current was observed (Figure 4D, WT: −70 mV = −0.12 ± 0.02; mutant: −70 mV = −0.48 ± 0.02). Furthermore, no difference in current density was recorded between WT and mutant channel [current density (pA/pF) for WT at +100 mV = 0.09 ± 0.02 and mutant at +100 mV = 0.08 ± 0.02], thus ruling out possible effects on trafficking of the channel. Both WT and mutant channels were blocked by the non-selective calcium channel blocker ruthenium red (Figures 4A and 4C). The increase in inward current was accompanied by a leftward shift in the midpoint (V1/2) of voltage activation curves derived from tail currents from +58.0 ± 2.1 mV in the WT channel to +1.7 ± 2.1 mV in the mutant channel (Figures 4E–4G). No change in voltage sensitivity was observed between WT and mutant channels (k = 52.3 ± 1.25 mV and 49.3 ± 1.56 mV for WT and mutant, respectively). Nevertheless, the small shift of the activation curves at negative potentials alone cannot account for the large increase in current in the mutant channel. This suggests that N855S mutation is also likely to affect the gating of TRPA1. In nominally Ca^2+^-free external solution, the TRPA1 half-activation voltages for WT and mutant channel were +83.3 ± 1.1 mV and +22.8 ± 1.0 mV, respectively (Figure S3). This shift in V1/2 between the WT and mutant channel was the same as in the presence of Ca^2+^ (∼60 mV), suggesting that external Ca^2+^ has no role in the shift of activation curves between WT and mutant channels. Nevertheless, in the absence of Ca^2+^ the slope of the voltage-activation curve of the mutant channel was shallower than the WT [k = 24.3 ± 1.8 mV and 42.2 ± 1.4 mV for WT and mutant, respectively (p < 0.01); Figure S3]. This demonstrates that the gating of the mutant channel is closely dependent on external Ca^2+^. It is worth noting that current traces at positive potentials always appear slower in the WT channel when compared to the mutant channel. This is similar to what is observed with heat-sensitized TRPV3 channels (Chung et al., 2005) and constitutes another argument in favor of a change in voltage dependence and gating of the channel by the N855S mutation. However, whether this has any physiological relevance is unlikely.

**Figure 5:**
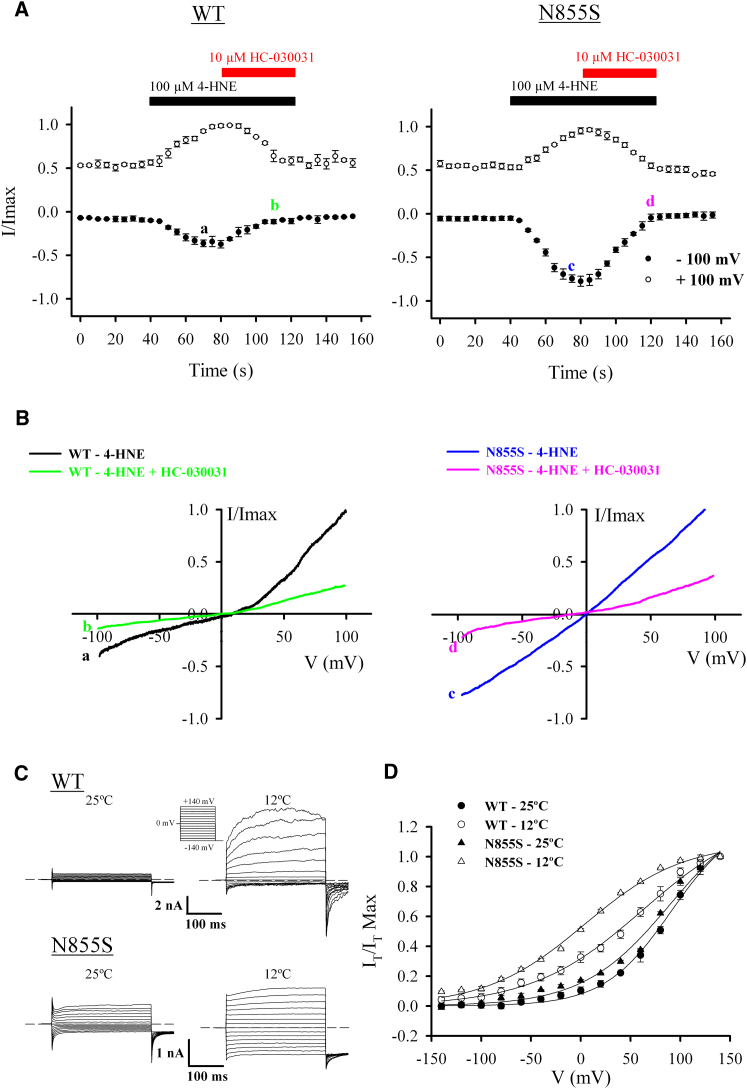
Activation of hTRPA1-WT and hTRPA1-N855S with an Endogenous TRPA1 Ligand and Cold (A) HEK293 cells expressing hTRPA1-WT (left; n = 5) or hTRPA1-N855S (right; n = 7) show activation by 4-HNE and block HC-030031. Currents were recorded at +100 mV and –100 mV and are normalized to current at +100 mV. Letters denote time point at which voltage ramps (shown in B) were acquired to generate current-voltage relationships. (B) Average current-voltage relationship of hTRPA1-WT and hTRPA1-N855S in the presence of 100 μM 4-HNE (as shown by letters a and c, respectively, in A) and after perfusion of 100 μM 4-HNE + 10 μM HC-030031 (as shown by letters b and d, respectively, in A). Currents are normalized to +100 mV. (C) Whole-cell current traces of HEK293 cells expressing hTRPA1-WT (top) or hTRPA1-N855S (bottom) in response to the indicated voltage step protocol applied at 25°C (left) and 12°C (right). Dotted line shows zero current level. (D) Mean steady-state activation curves obtained from tail currents (I_T_) at −140 mV for hTRPA1-WT (n = 5) and hTRPA1-N855S (n = 5) in response to 25°C and 12°C. Error bars in all plots represent SEM across individual cell measurements.

The enhanced activity in the mutant channel was also apparent when the ligand used was the endogenous mediator 4-hydroxynonenal (4-HNE; Figures 5A and 5B), as well as mustard oil (Allyl isothiocyanate; Figure S2B) or menthol (Figure S4). Both WT and mutant channel types were blocked by the selective antagonist HC-030031 (Figures 5A and 5B).

The mechanism of activation of TRPA1 by cold is a contentious subject. Here we show that cooling activated both WT and mutant channels with increased inward currents associated with the N855S variant (Figure 5C). This is due to a leftward shift of the activation curve with the mutant channel showing a greater shift compared to the WT channel (V1/2 = +90.9 ± 1.3 mV and +62.7 ± 1.6 mV at 25°C and 12°C, respectively, for WT and V1/2 = +90.3 ± 1.1 mV and +4.0 ± 1.8 mV at 25°C and 12°C, respectively, for the mutant channel; Figure 5D). This effect was reversed upon application of the TRPA1-specific antagonist HC-030031 (Figure S5). This shift in voltage dependence of channel activation toward more negative potentials is similar to what has been described for other temperature-sensitive TRP channels (Voets et al., 2004). The increase in inward current in the mutant channel is therefore independent of activation mechanism and is observed with all ligands tested as well as with cold activation (Table S5).

## Discussion

The present study unambiguously identifies a gain-of-function point mutation in *TRPA1* (N855S) as the cause of the previously undescribed human pain syndrome, which we have named FEPS. This mutation is highly penetrant, giving rise to stereotyped episodes of severe pain affecting principally the upper body triggered by cold and fasting; there is complete segregation of the mutation with the clinical syndrome. Biophysical studies using heterologously expressed channels show that the N855S mutation does not alter exogenous or endogenous ligand binding, but does increase current flow through the activated channel at negative membrane potentials.

FEPS is likely to be rare as it has not previously been described in the pain literature. A number of conditions such as Fabry's disease (Zarate and Hopkin, 2008) or familial amyloid neuropathy (Wang et al., 2008), which result in injury to nociceptor axons (small fiber neuropathies), are associated with neuropathic pain in the extremities. In FEPS patients, however, the pain has a proximal distribution, is episodic, and intraepidermal nerve fiber density [a sensitive measure of small fiber neuropathy (Lauria et al., 2005)] was normal. Therefore, in a manner analogous to gain-of-function mutations in Na_V_1.7 (Yang et al., 2004, Fertleman et al., 2006, Drenth and Waxman, 2007), FEPS occurs as a consequence of altered functional properties of nociceptive afferents. In these patients we did not observe any changes in baseline mechanical or thermal pain thresholds. However, there was evidence of enhanced sensitization of the nociceptive system following application of mustard oil (a TRPA1 agonist). The greater flare response reflects increased neurogenic inflammation and the increase in the area of punctate hyperalgesia probably represents enhanced central sensitization due to increased nociceptor drive. The initial trigger for TRPA1 activation is unknown, but the contribution of tiredness, cold, and fasting to the attacks, coupled with the preventive role of food and warming, suggests that some metabolic dysfunction, for example, lowered membrane potentials or increased intracellular calcium levels, could contribute to the start of attacks (Zhang and Lipton, 1999, Velasco et al., 2006).

The molecular basis of FEPS resides in the N855S mutation present adjacent to a cysteine residue in the putative S4 domain of TRPA1. Activation of TRPA1 by electrophilic compounds, such as 4-HNE and mustard oil, has been shown to occur through covalent modification of N-terminal cysteines (Macpherson et al., 2007, Peterlin et al., 2007, Trevisani et al., 2007, Materazzi et al., 2008, Taylor-Clark et al., 2009). In contrast, TRPA1 activation by non-electrophilic compounds such as menthol is determined through transmembrane domain 5 (Xiao et al., 2008). The present studies suggest that agonist binding to the mutant channel remains unaffected. However, the N855S mutant has complex effects on channel behavior, mixing a shift of TRPA1 activation toward more negative voltages and a change in the gating of TRPA1 through a Ca^2+^-dependent mechanism. Previous studies have shown that the voltage dependence of thermoTRPs is linked to the S4 segment (Voets et al., 2004, Voets et al., 2007). The present results demonstrate that the S4 transmembrane segment of TRPA1 contributes to agonist- and temperature-dependent channel activity. A similar mutation in the S4 segment of TRPM8 at an adjacent amino acid residue (856) also resulted in a leftward shift in the voltage activation curve (Voets et al., 2007). This suggests that temperature and ligand regulation of voltage dependency among thermoTRPs may be conserved.

Our data also suggest that the S4 segment of TRPA1 is involved in gating the channel via a Ca^2+^-dependent mechanism. Internal Ca^2+^-dependent activation of TRPA1 was shown to occur through an EF-hand domain in the N terminus of the channel (Zurborg et al., 2007). Here we show that external Ca^2+^ regulates the voltage sensitivity of TRPA1 through the S4 segment of the channel. This is the first demonstration of an effect of Ca^2+^ mediated by the S4 segment of a TRP channel. A fascinating aspect of TRPA1 activation lies in interspecies discrepancies. Some electrophilic thioaminal-containing compounds are able to activate rat TRPA1 while others inhibit human TRPA1 (Chen et al., 2008). This difference is due to key amino acids situated in the S6 segment. A similar story applies to caffeine, which was shown to activate mouse TRPA1 but to inhibit the human channel (Nagatomo and Kubo, 2008), and menthol, which activates mammalian TRPA1, blocks nonmammalian TRPA1 (Xiao et al., 2008), and exerts a bimodal action on murine TRPA1 (Karashima et al., 2009). Therefore, similar compounds are able to exert opposite effects in different species and this suggests a complex gating mechanism for TRPA1 in which amino acid substitutions at key positions determine agonist binding and gating. The N855S mutation induces a linearization of TRPA1's current-voltage relationship, as can be observed with heat-sensitized TRPV3 channels (Chung et al., 2005). The similarities with sensitized TRPV3 extend to the leftward shift of the voltage dependence and weaker time-dependent current increase at depolarizing potentials. In contrast, while mutant TRPA1 channels have the same reversal potential as the WT, suggesting no change in ion permeability, sensitized TRPV3 has an altered reversal potential, reflecting a change in ion permeability (Chung et al., 2005). This suggests that different mechanisms underlie the linearization of voltage dependence in TRPA1 and TRPV3. This view is strengthened by the fact that removing external Ca^2+^ increases TRPV3 current amplitude, whereas it shifts activation curves of both WT and N855S TRPA1 toward more depolarized potentials while at the same time decreasing N855S TRPA1 voltage sensitivity.

In this study we also provide further evidence that TRPA1 can be gated by cold. Cold was first proposed not to be a direct activator of TRPA1 but rather a trigger for an increase in intracellular [Ca^2+^] leading to downstream activation of TRPA1 (Zurborg et al., 2007). This view has been recently challenged with the demonstration that cold is able to activate TRPA1 in the absence of Ca^2+^ both inside and outside the cell (Karashima et al., 2009). Here we confirm that TRPA1 can be activated by cold and the mutant channel shows a similar gain of function on cold application to that observed with chemical ligands (Table S5). The evidence of a role for TRPA1 in transducing cold pain is strong, while a contribution to mechanosensation may be downstream of primary mechanotransducers (Kwan et al., 2006, Kwan et al., 2009, Bautista et al., 2006). The enhanced currents seen with application of cold temperatures in the mutant channel are consistent with a possible role for TRPA1 as a cold sensor, although there is no doubt that cold sensors other than TRPA1 are expressed by sensory neurons (Munns et al., 2007, Kwan et al., 2009, Karashima et al., 2009). Finally, the demonstration that mutant channels are sensitive to HC-030031 suggests that specific TRPA1 antagonists may have a useful therapeutic role in this pain syndrome (Eid et al., 2008).

Enhanced channel activity associated with the N855S mutation is thus consistent with the pain syndrome observed in FEPS patients. Increased activity of the mutant channel when activated by endogenous mediators provides a plausible mechanism that could explain the intense pain experienced by carriers of the N855S mutation, while the localized effect may reflect high levels of channel expression or the site of production of activating ligands. There is evidence that SNP variants in *TRPA1* influence differential sensitivity to experimentally induced cold pain in humans (Kim et al., 2006). Our results provide both a mechanism and a therapeutic approach to treat the pain episodes experienced in FEPS, which is the first pain-related TRP channelopathy to be described in humans. It will be of great interest to establish whether TRPA1 channel variants or misregulation contribute to the risk and severity of chronic pain in patient populations.

## Experimental Procedures

### Study Subjects

The family studied was identified in Antioquia, in North-West Colombia (Bedoya et al., 2006). Unaffected controls used for screening of the A2564G mutation were also ascertained in Antioquia. This study was approved by the ethics committee of the Universidad de Antioquia and was compliant with the Declaration of Helsinki 2008. Written informed consent was given by all study subjects.

### Microsatellite Typing and Linkage Analysis

A whole-genome scan using 552 microsatellite markers with an average intermarker distance of 8 cM was performed in 13 affected and 10 unaffected members of the FEPS family (Figure 1). Genotyping was carried out by deCODE Genetics. Parametric linkage analysis was performed using LINKAGE (Lathrop et al., 1984, Lathrop et al., 1986, Lathrop and Lalouel, 1984) (two-point) and SimWalk2 (Sobel and Lange, 1996) (multipoint). Penetrances were set to 0.985 for both homozygous and heterozygous carriers. The phenocopy rate was set to 0 and the disease allele frequency to 0.01%. Maximum likelihood haplotype reconstruction was performed using SimWalk2. For fine-mapping, an additional 15 microsatellite markers (D8S533, D8S1767, D8S1775, D8S1792, D8S1117, D8S543, D8S1795, D8S1807, D8S1776, D8S275, D8S1988, D8S1822, D8S276, D8S85, and D8S1122) were genotyped in all 23 individuals across the initial region of the linkage signal on chromosome 8q12.1–8q24.1 (Figure 1). Genotyping was carried out by PCR using fluorescence-labeled primers and standard reaction conditions followed by fragment length analysis on an ABI3037*xl* Genetic Analyzer (Applied Biosystems). After fine mapping, multipoint linkage and haplotype analyses were repeated as described above.

### Candidate Gene Sequencing

After fine mapping, haplotype analysis defined a candidate region delimited by markers D8S1775 and D8S276 on chromosome 8q13.2–8q22.2. Genes within this region were identified using the BioMart data mining tool (http://www.biomart.org) on build 35.1 of the human genome sequence. Among the 287 genes in the region, the following candidate genes were chosen based on their potential roles in excitability and pain signaling: proenkephalin (*PENK*), the cation channel *TRPA1*, and potassium channel genes *KCN B2* and *KCN S2*. A further potassium channel gene, *KCN V1*, though outside the narrow candidate region, was located within the initial linkage peak on chromosome 8q23.2 and was therefore included in candidate gene sequencing.

Amplicons covering exons and intron/exon boundaries, as well as the promoter regions (∼1 kb upstream of the start of translation), were designed using Primer3 (http://frodo.wi.mit.edu/; primer sequences are available upon request), and all amplicons were sequenced bidirectionally in at least one affected and one unaffected individual using standard dideoxy sequencing on an ABI 3730*xl* Genetic Analyzer (Applied Biosystems). Exon 22 of the *TRPA1* gene was sequenced in a similar manner in 13 affected and 9 unaffected members of the Antioquian family (to check for cosegregation of the A2565G mutation with the phenotype) and in 139 Antioquian population controls.

### Biophysical Studies of TRPA1

A full-length coding sequence of *TRPA1* was amplified from IMAGE clone 100015422 (BC148423; Geneservice) using the forward primer (5′-CCCCAAGCTTTCCGGGGTGGGGTCAATGAAGCGCAGCCTGAGGAAGAT) and the reverse primer (5′-CCGCTCGAGCGGATTAGAAGCCTCACTGAAGGTCTGAGGAGCTAAGGCTCAAGATGGTGTGTTTTTG). This 3426 bp PCR product was then digested with HINDIII and XhoI and ligated into clone pcDNA3JCPOLRED to give the final clone TRPA1RED. The final clone (TRPA1RED) was sequenced entirely and corresponds to *TRPA1* RefSeq sequence NM_007332. The clone TRPA1RED was used as a template to generate the c.A2564G mutation using the QuikChange XL Site-Directed Mutagenesis Kit (Stratagene) according to the manufacturer's instructions. This clone was sequenced entirely by standard methods.

HEK293 cells were transfected with cDNA clones using Lipofectamine 2000. Intracellular free calcium was measured using dual excitation of the calcium-sensitive fluorescence probe Fura-2 (Molecular Probes). Patch clamp electrophysiological recordings were performed using an Axopatch 200B patch-clamp amplifier (Molecular Devices). Full details of transfection, calcium imaging, and recording protocols are to be found in Supplemental Information.

### Quantitative Sensory Testing

QST was performed on nine patients with FEPS as well as in eight unaffected siblings who did not carry the mutation and who were matched as far as possible for age and sex. None of the subjects had comorbid medical conditions (such as diabetes), which could impair sensory function, were taking medication, or were experiencing ongoing pain at the time QST was performed. The experimenter was blind to the subjects' genotype. Vibration detection threshold was measured using a 128 Hz Rydel-Seiffer tuning fork placed on the distal phalanx of the index finger. Three readings were taken and the mean was calculated. The mechanical detection threshold was determined using von Frey hairs (0.06 to 644 mN). Five threshold determinations were determined using the “method of limits” with ascending and descending stimulus intensities and the final threshold was the geometric mean of these five series. Pressure pain threshold was determined over the ulnar eminence using a pressure gauge (FDN100; Wagner instruments USA; probe area of 1 cm^2^ up to 1000 kPa). Thermal thresholds were determined on the volar forearm using a 16 ×16 mm probe held at an adaption temperature of 32°C connected to a servo-controlled Peltier device (TSA-II; Medoc). Thresholds were obtained with a ramp stimulus (1°C/S) that terminated when the subject pressed a button at which point probe temperature rapidly returned to the adaption temperature of 32°C. The mean of three readings was taken. Cold pressor pain threshold was determined by measuring the latency to the first pain sensation after immersion of the hand up to the level of the wrist in ice water kept at 4°C. This was repeated three times with at least 2 min between each test (Martikainen et al., 2004).

### Mustard Oil-Evoked Sensitization

200 μl of 50% mustard oil (Allyl isothiocyanate; Fluka; v/v in olive oil) was applied to a 0.64 cm^2^ region of the volar forearm for 10 min. During this period pain scores were recorded using electronic VAS. An acetate template was used to mark dots at 1 cm increments along eight spokes radiating out from the area of mustard oil application. 10, 30, and 60 min after mustard oil application, sensory testing was performed starting at the outermost spoke to act as reference and moving toward the area of mustard oil application. Punctate hyperalgesia was determined using a 100 mN filament (Bailey Instruments) applied once and brush evoked allodynia using a No. 2 sable paintbrush (Justbrushes) applying four strokes of 1 cm perpendicularly to the spoke at each point. The area of flare, punctate hyperalgesia, and brush-evoked allodynia was determined as per Norbury et al. (2007). The primary area of mustard oil application was subtracted from these figures to determine the area of secondary change for brush-evoked allodynia and punctate hyperalgesia.

### Determination of Intraepidermal Nerve Fiber Density

3 mm punch skin biopsies were taken from the upper arm, a commonly affected region during pain. Skin was immersion fixed in 4% paraformaldehyde overnight and then transferred to 20% sucrose in 0.1 M phosphate buffer for 24 hr. 50 μm free floating sections were cut and immunostained using an antibody directed against the pan-neuronal marker PGP 9.5 (1:1000 ultraclone), and intraepidermal nerve fiber density was calculated as per Lauria et al. (2005).

## Supplemental Information

Document S1. Supplemental Figures, Tables, and Supplemental Experimental Procedures
