# Supplementary figures and images for: A Gain-of-Function Mutation in TRPA1 Causes Familial Episodic Pain Syndrome
Source: Neuron. 2010 Jun 10;66(5):671–80. doi: 10.1016/j.neuron.2010.04.030 (PMC4769261; doi:10.1016/j.neuron.2010.04.030)

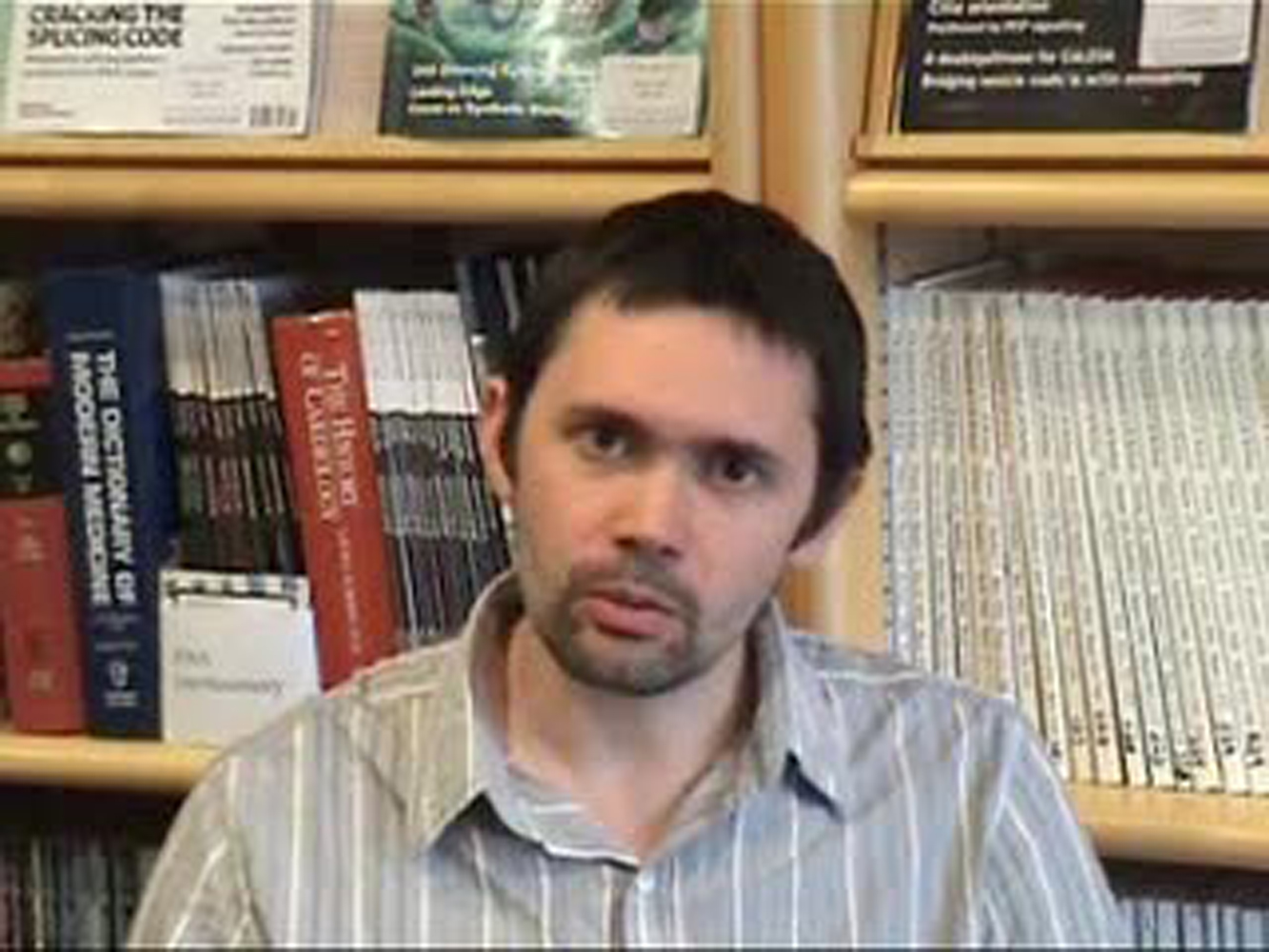

Supplement: Supplementary file 1 [file mmc2.jpg]
