# Supplementary material for: A Gain-of-Function Mutation in TRPA1 Causes Familial Episodic Pain Syndrome
Source: Neuron. 2010 Jun 10;66(5):671–80. doi: 10.1016/j.neuron.2010.04.030 (PMC4769261; doi:10.1016/j.neuron.2010.04.030)
Supplement: Document S1. Supplemental Figures, Tables, and Supplemental Experimental Procedures [file mmc1.pdf]

Neuron, Volume 66 Supplemental Information

A gain-of-function mutation in TRPA1 causes Familial Episodic Pain Syndrome

Barbara Kremeyer, Francisco Lopera, James J. Cox, Aliakmal Momin, Francois Rugiero, Steve Marsh, C. Geoffrey Woods, Nicholas G. Jones, Kathryn J. Paterson, Florence R. Fricker, Andrés Villegas, Natalia Acosta, Nicolás G. Pineda-Trujillo, Juan Diego Ramírez, Julián Zea, Mari-Wyn Burley, Gabriel Bedoya, David L.H.Bennett, John N. Wood, and Andrés Ruiz-Linares

Table S1. Summary of the clinical characteristics of 17 patients suffering from FEPS.

Characteristic	n	Freq	Mean (±stdev)	Range
**Demographic**				
Sex (M:F)	17	8:9 (47:53)		
Present Age (years)	17		34.5 (20.7)	11-70
**Clinical Features:**				
Age of Onset (months)	16		7.25 (8.1)	1-36
Age at last episode (years)	17		22.3 (18)	3-69
Number of episodes	14		31.2 (17.5)	5-60
Time from onset to max pain (minutes)	14		13.2 (14.0)	5-60
Max duration (minutes)	17		62.7 (12.1)	45-90
Min duration (minutes)	17		34.4 (12.1)	5-60
Most freq episode in childhood Y:N	17	17 (100%)		
Severity (0-10, arbitrary units)	17		9.7 (0.6)	8-10
**Triggers:**<br>Fasting<br>Cold<br>Physical exercise<br>Illness	17	 17 (100%)<br>12 (70.6%)<br>12 (70.6%)<br>8 (47.1%)		
**Location of pain:**<br>Shoulder and/or arms<br>Thorax<br>Abdomen<br>Cervical spine<br>Whole body	17	 8 (47%)<br>6 (35%)<br>2 (12%)<br>2 (12%)<br>2(12%)		
**Measures to avoid progression**<br>Eating<br>Warming of the body<br>Resting	17	 17 (100%)<br>9 (53%)<br>4 (23%)		
**Accompanying symptoms/signs**<br>Pallor<br>Perspiration<br>Peribuccal cyanosis<br>Nausea and/or vomiting	17	 16 (94%)<br>16 (94%)<br>14 (82%)<br>8 (47%)		
**Features following an episode**<br>Somnolence<br>Fatigue<br>Anhedonia<br>Confusion	17	 14 (82%)<br>9 (53%)<br>5(29%)<br>6 (%)		

Table S2. Results of McGill pain questionnaire Spanish version (MPQ-SV)(*10*) parameters in FEPS patients (n=9). Abbreviations: PRI: Pain Rating Index; T: Total, S: Sensory, A: Affective, E: Evaluative.

**MPQ-SV parameter**	Score mean (±SD)	Range
PRI-T	31.6 (±5.2)	11-49
PRI-S	20.8 (5.2)	8-40
PRI-A	6.8 (1.1)	0-9
PRI-E	4 (0)	0-4

Table S3. Descriptors of MPQ-SV chosen by at least 40% 0f FEPS patients to describe pain episodes (both Spanish term and English translation are shown)

MPQ SV Descriptor-Spanish	MPQ SV Descriptor (English translation)	% of patients
Terriblemente Molesto	Unbearable	100
Pesadez	Heavy	66
Que amarga la vida	Making life bitter	66
Aterrador	Terrifying	66
Periodico	Periodic	55
Repartido (en una zona)	Spread (to an area)	55
Como un peso	Like a weight	55
A golpes	Thumping	44
Continuo	Continuous	44
Impreciso	Poorly localised	44
Extenso	Extensive	44

Table S4: A comparison of QST findings in family members, with and without FEPS. Data expressed as mean (standard deviation) unless stated otherwise. * For these values n=6 and 3 for FEPS and control respectively. Statistics § Unpaired t-test, † Fisher's exact test, ‡ Mann-Whitney Rank Sum Test.

	FEPS (n=9)	Control (n=8)	*P*-value
Age (years)	37 (21)	34 (18)	0.69§
Gender (Male:Female)	4:5	4:4	1.00†
Heat pain threshold (^{o}C)*	45.0 (3.6)	48 (2.4)	0.26§
Cold pain threshold (^{o}C)*	6.6 (9.1)	7.97 (5.8)	0.83§
Cold pressor pain threshold (seconds)	33.0 (39.6)	11.2 (4)	0.17‡
Pressure pain threshold (kPa)	558 (140)	580 (171)	0.77§
Vibration detection threshold (arbitrary units)	7.7/8 (0.37)	7.7/8 (0.21)	0.53‡
Tactile detection threshold (mN)	1.9 (1.2)	1.8 (0.5)	0.74‡

Table S5 Summary of the biophysical characterisation of hTRPA1-WT and hTRPA1-N855S showing mid-point for voltage activation curves (V½), slope factor (k value) and ratio of inward to outward current in response to agonists from -100mV to +100mV respectively. CA = cinnamaldehyde, 4-HNE = 4-Hydroxynonenal and AITC = Allyl isothiocynate. Data are shown as mean ± S.E.M. † = P<0.001

	**V½ (mV)**		**k value (mV)**		**Ratio of inward to outward current**	
Agonist	*WT*	*N855S*	*WT*	*N855S*	*WT*	*N855S*
CA	+ 58.0 ± 2.1	+ 1.7 ± 2.1 †	52.3 ± 1.3	49.3 ± 1.6	0.15 ± 0.02	0.70 ± 0.02
CA (no Ca^{2+})	+ 83.3 ± 1.1	+ 22.8 ± 1.0 †	24.3 ± 1.8	42.2 ± 1.4 †	0.06 ± 0.01	0.62 ± 0.01
4-HNE	+ 55.3 ± 1.9	+ 5.1 ± 1.8 †	48.2 ± 1.9	45.3 ± 1.7	0.19 ± 0.02	0.72 ± 0.01
AITC	+ 59.6 ± 2.2	+ 1.3 ± 2.2 †	51.3 ± 2.4	49.1 ± 2.1	0.61 ± 0.03	1.24 ± 0.03
Menthol	+ 57.5 ± 1.8	+ 4.8 ± 1.6 †	48.6 ± 1.8	52.3 ± 2.1	0.09 ± 0.02	1.00 ± 0.03
12°C	+ 62.7 ± 1.6	+ 4.0 ± 1.8 †	56.0 ± 1.2	49.6 ± 2.3	0.05 ± 0.01	1.42 ± 0.02

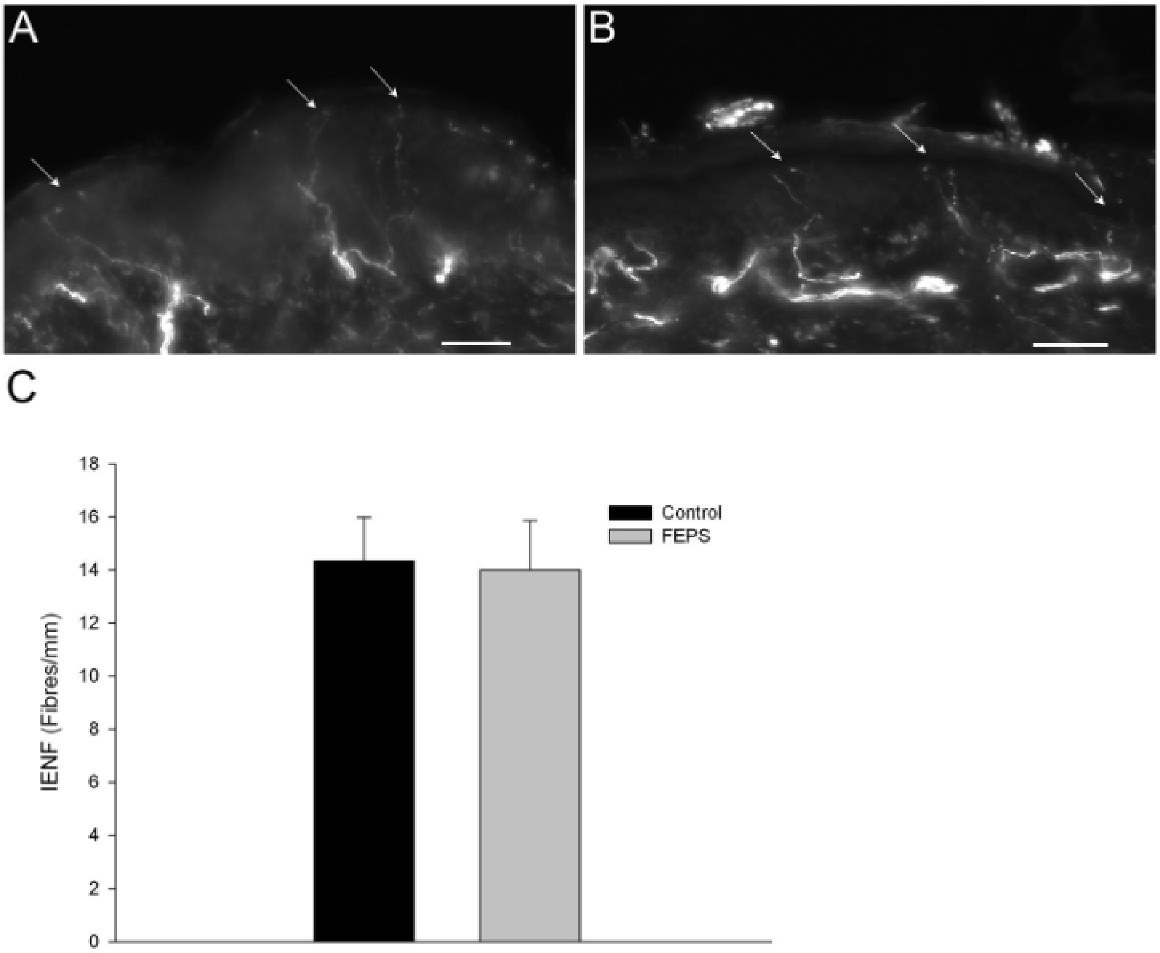

Figure S1. Intra-Epidermal Nerve Fibres (IENF) were visualised by immunostaining with the pan-neuronal marker PGP 9.5 (arrows). These show normal morphology in FEPS patients (B) compared to non-affected siblings (A) and there was no change in IENF density. Scale bar represents 100 μm.

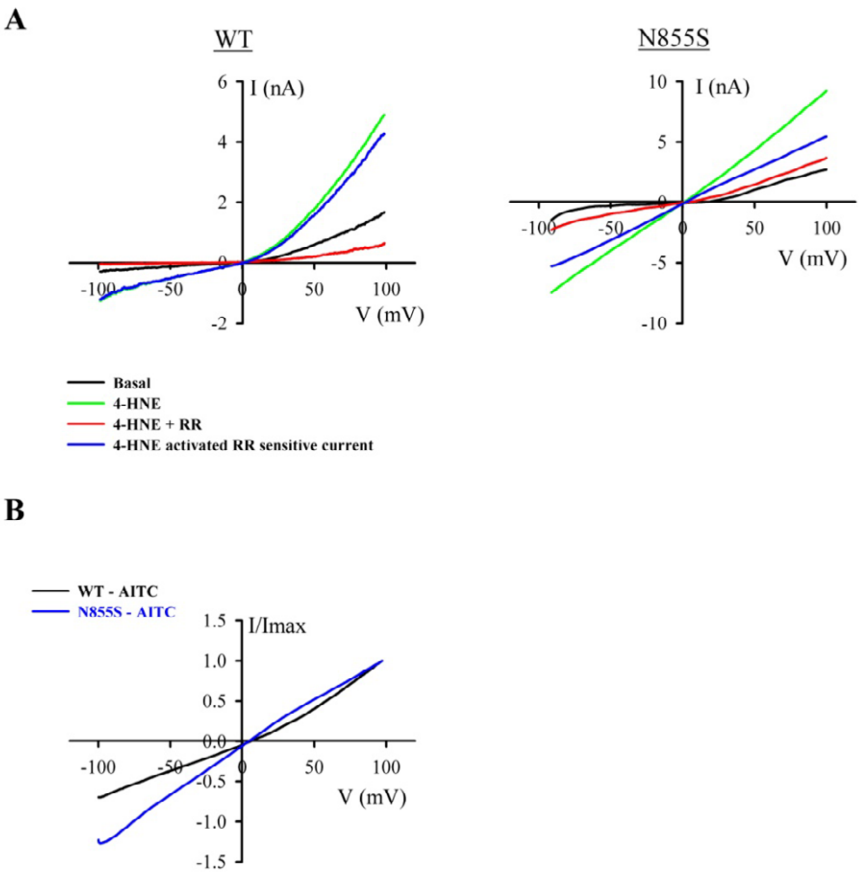

Figure S2. Response of hTRPA1-WT and hTRPA1-N855S with endogenous and exogenous TRPA1 ligands. (A) Representative current-voltage relationship of hTRPA1-WT (left panel) and hTRPA1-N855S (right panel). Black trace, basal current before 4-hydroxynonenal (4-HNE) application; green trace, fully activated currents after 100 μM 4-HNE application; red trace, ruthenium red (RR, 3 μM) inhibitor of 4-HNE activated currents; blue trace, 4-HNE activated ruthenium red sensitive current obtained by subtracting red trace from green trace. **(B)** Average current-voltage relationship of hTRPA1-WT (n = 7) and hTRPA1-N855S (n = 5) in the presence of mustard oil (100 μM allyl isothiocyanate, AITC). Currents are normalised to +100 mV.

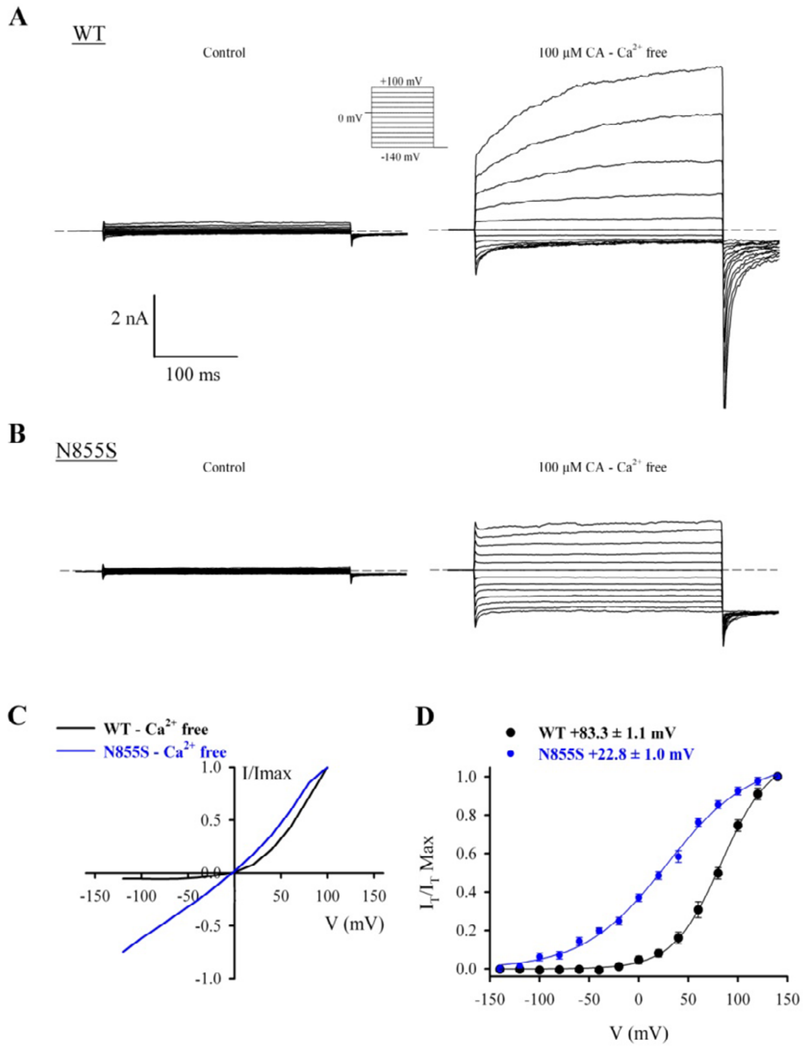

Figure S3. Response of hTRPA-WT and hTRPA1-N855S in the presence of extracellular Ca^{2+} free extracellular solution. (A) Whole-cell current traces of HEK293 cells expressing hTRPA1-WT in response to the indicated voltage step protocol in the absence (left panel) and presence (right panel) of 100 μM CA in Ca^{2+}-free extracellular solution. Dotted line shows zero current level. **(B)** Same as **A** but in HEK293 cells expressing hTRPA1-N855S. **(C)** Average current-voltage relationship of hTRPA1-WT (n = 4) and hTRPA1-N855S (n = 6) in the presence of 100μM CA in Ca2+-free intracellular and extracellular solution. Currents are normalised to

+100 mV. (D) Mean steady-state activation curves derived from tail currents (IT) at -140 mV for hTRPA1-WT and hTRPA1-N855S.

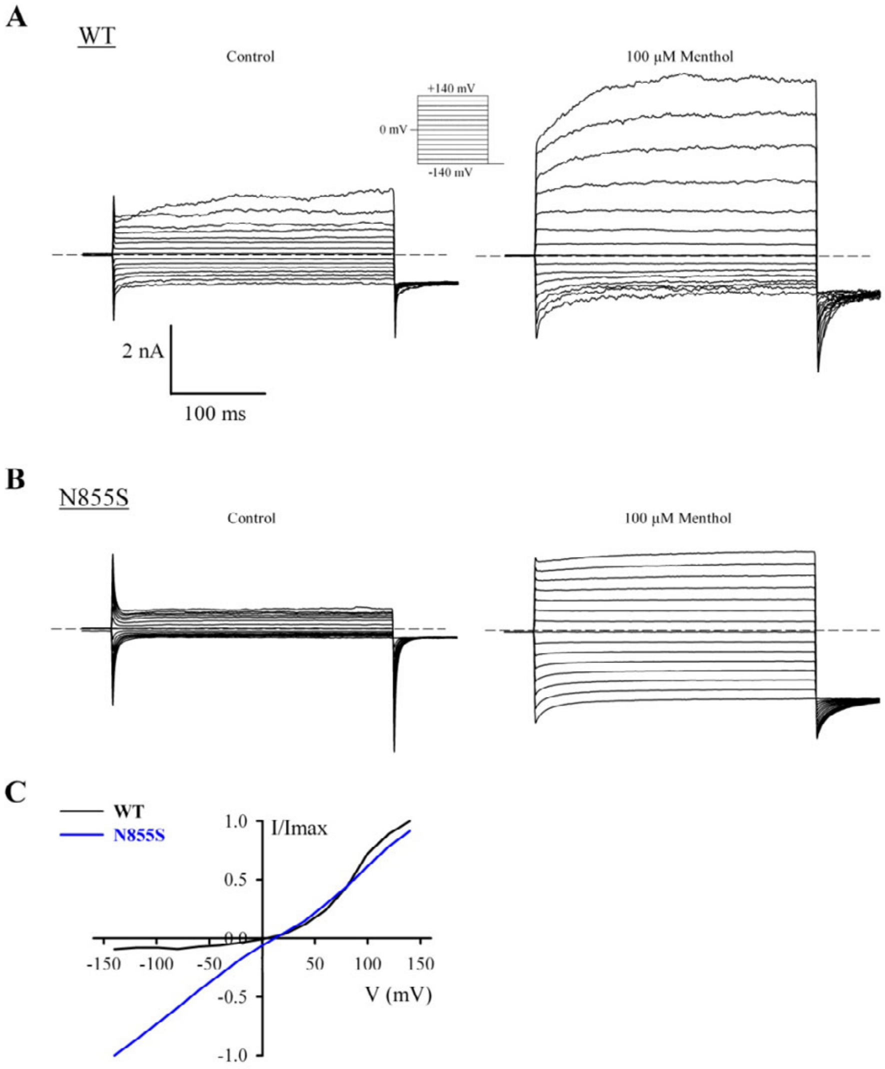

Figure S4. Response of hTRPA1-WT and hTRPA-N885S with menthol. (A) Whole-cell current traces of HEK293 cells expressing hTRPA1-WT in response to the indicated voltage step protocol in the absence (left panel) and presence (right panel) of 100 μM Menthol. Dotted line shows zero current level. **(B)** Same as **A** but in HEK293 cells expressing hTRPA1-N855S. **(C)**

Average current-voltage relationship of hTRPA1-WT (n = 5) and hTRPA1-N855S (n = 5) in the presence of 100 μM Menthol. Currents are normalised to +140 mV.

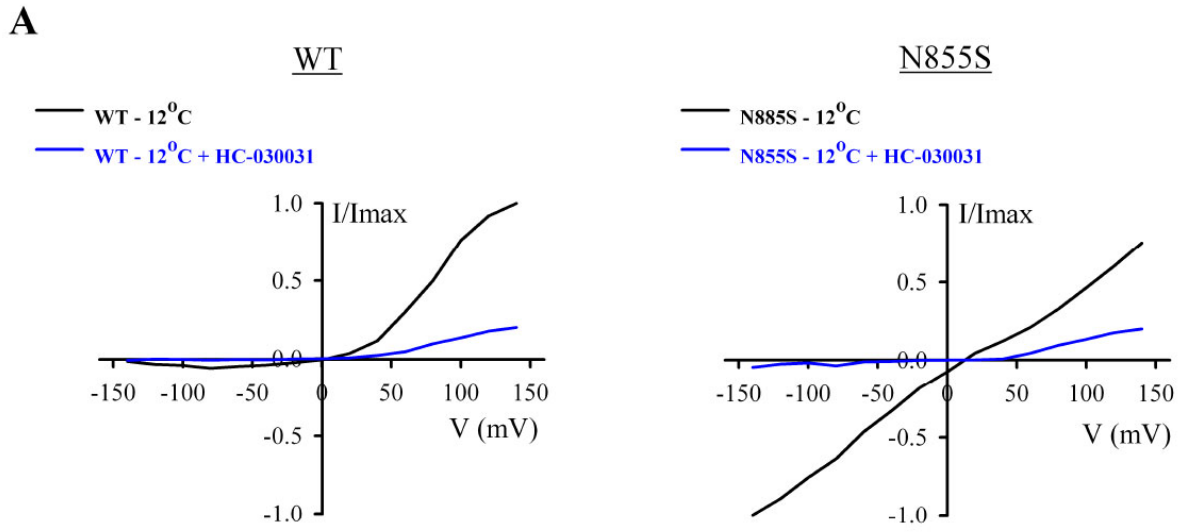

Figure S5. I/V relationship of hTRPA1-WT and hTRPA1-N855S in response to cold.

Average current-voltage relationship of hTRPA1-WT (left panel, n = 5) and TRPA1-N855S (right panel, n= 5) in response to 12°C and in response to 12°C + HC-030031.

List of URLs

The UCSC Human Genome Browser is available at

http://genome.ucsc.edu/cgi-bin/hgGateway;

BLAST is available at http://www.ncbi.nlm.nih.gov/BLAST.

The BioMart tool is available at http://www.biomart.org.

Primer3 is available at http://frodo.wi.mit.edu/.

Supplemental Experimental Procedures

Clinical characteristics of Familial Episodic Pain Syndrome

Below we describe an illustrative case history of a patient with FEPS. Table S1 summarises the clinical characteristics of 17 patients with FEPS. We can find no report of a similar inherited pain syndrome in the literature. Tables S2 and S3 demonstrate the findings of the Spanish Version (SV) of the McGill pain questionnaire. Table S4 shows the findings on Quantitative Sensory Testing (QST).

Case history

JG is currently 20 years old. Her birth and early development were normal. At the age of 7 months her mother noted periods of distress and prolonged crying with no apparent cause. She describes episodes of severe pain which can be triggered by fasting, exercise and exposure to cold during childhood. She would first note pain in a symmetrical distribution in the upper arms, as well as anterior and posterior chest wall. The pain was described as aching and pressing and would progressively increase in intensity (scoring 10 on a 0-10 numerical rating scale). Within the first 15 minutes she may be able to terminate an attack by eating and warming herself (for

instance wearing blankets if she has been swimming). The pain would normally reach maximal intensity at 45 minutes following onset. She has only ever taken simple analgesia in the form of non-steroidal anti inflammatory drugs which are ineffective. During an episode her family describe her as being pale and perspiring and she also describes an increase in her heart rate. She usually lies down whilst experiencing pain. The total duration of the pain is approximately an hour and she then sleeps; upon waking she is pain free. She has experienced these episodes up to a frequency of once per week in childhood. She has not experienced a severe episode in the last 6 years as she is able to avoid the triggers and terminate an episode when she first experiences pain. Between these episodes sensory function is normal she denies any numbness, paraesthesia or altered pain sensibility. Cognitive function is normal and she is currently a student. Her father, younger brother and sister also suffer from the same episodic pain syndrome. She has no other past medical history of note and she takes no regular medication.

TRPA1 Cloning

To aid in the identification of positively transfected cells, the constructs were designed so that the red fluorescent protein, DsRed2, was translated separately to TRPA1 from an internal ribosome entry site (IRES). The wild-type *TRPA1* ➔ polio IRES ➔ *DsRed2* construct was made as follows. The polio IRES-*DsRed2* fragment (1487 bp) was PCR-amplified from vector POLRED1 (Cox et al., 2006) using the forward primer (5' CCG CTC GAG CGG CCG CTA GCG CTA CCG GAC TC) and the reverse primer (5' CTA GAG GGC CCC TAC AGG AAC AGG TGG TGG C) and Phusion High-Fidelity DNA Polymerase (New England Biolabs). This fragment was digested with *Xho*I and *Apa*I and ligated into the low-copy number mammalian expression vector pcDNA3JC (Cox et al., 2006) to give clone pcDNA3JCPOLRED. Next, the full-length

coding sequence of *TRPA1* was amplified from IMAGE clone 100015422 (BC148423; Geneservice) using the forward primer (5' CCC CAA GCT TTC CGG GGT GGG GTC AAT GAA GCG CAG CCT GAG GAA GAT) and the reverse primer (5' CCG CTC GAG CGG ATT AGA AGC CTC ACT GAA GGT CTG AGG AGC TAA GGC TCA AGA TGG TGT GTT TTT G). This 3426 bp PCR product was then digested with *HIND*III and *Xho*I and ligated into clone pcDNA3JCPOLRED to give the final clone TRPA1RED. For these cloning steps the ligation reactions were initially transformed into XL-10 Gold Ultracompetent Cells (Stratagene) and single colonies grown up for isolation of DNA using the QIAprep Spin Miniprep Kit (Qiagen). A DNA aliquot was checked by restriction digest and then plasmid DNA was transformed into SURE2 Supercompetent Cells (Stratagene). Resultant single colonies were grown up for DNA isolation using the HiSpeed Plasmid Midi Kit (Qiagen). The final clone (TRPA1RED) was sequenced entirely and corresponds to *TRPA1* RefSeq sequence NM_007332. The clone TRPA1RED was used as template to generate the c.A2564G mutation using the QuikChange XL Site-Directed Mutagenesis Kit (Stratagene) according to the manufacturer's instructions. This clone was sequenced entirely by standard methods.

Cell culture and transfection

HEK-293 cells were cultured in DMEM and 10 % Foetal Bovine Serum (FBS). Cells were co-transfected with pMaxGFP (Amaxa) and either human TRPA1 wild-type or human TRPA1-N855S in the pcDNA3 expression plasmid with Lipofectamine 2000 (Invitrogen) at a ratio of 1:5. For intracellular Calcium imaging, cells were co-transfected with Ds-Red-monomer fluorescent protein (Clontech) and either human TRPA1 wild-type or hTRPA1-N855S at a ratio of 1:1. The total amount of plasmid DNA transfected was 1μg/ul per 35 mm dish.

Patch-clamp recordings

Extracellular solution contained (in mM): 140 NaCl, 4 KCl, 2 $CaCl_2$, 1 $MgCl_2$, 10 HEPES, adjusted to pH 7.4 with NaOH, osmolarity 300–310 mosmol l^{-1}. Pipettes were filled with an intracellular solution containing (in mM): 130 KCl, 8 NaCl, 2 EGTA, 1 $MgCl_2$, 1 $CaCl_2$, 4 MgATP, 0.4 Na_2GTP, adjusted to pH 7.4 with CsOH, osmolarity 300–310 mosmol l^{-1}. For experiments performed in Ca^{2+}-free conditions, Ca^{2+} was substituted with 3 mM NMDG in the extracellular solution.

Experiments were performed using an Axopatch 200B patch-clamp amplifier (Molecular Devices). Currents were recorded in response to a voltage ramp from -100 mV to + 100 mV for 180 milliseconds every 2 seconds. Voltage dependence was determined by measuring currents during a voltage step protocol consisting of 300-ms voltage steps to test potentials ranging from -140 mV to + 140 mV, followed by a final step to -140 mV to measure tail currents. Tail current derived voltage activation curves were fitted to the Boltzmann equation of the following form:
$I_T/I_{T\,(Max)} = 1/(1 + \exp[(V - V_{½})/k])$, where V is the membrane potential of the prepulse, $V_{½}$ is the membrane potential at which the current is half activated, k is the slope factor, I_T is the current amplitude of the tail current recorded for a given prepulse and $I_{T(Max)}$ is the maximum current amplitude of the tail current. Whole-cell membrane currents were filtered at 5 kHz and sampled at 10 kHz. Data were acquired and analysed using pCLAMP 9.2 software (Molecular Devices). All recordings were made at room temperature unless stated.

Intracellular Calcium imaging

Intracellular free calcium $[Ca^{2+}]_I$ was measured using dual excitation of the calcium-sensitive fluorescence probe Fura-2 (Molecular Probes). Fura-2 was loaded into the cell by using the acetoxymethyl-ester form (Fura-AM, 3 μM, 60 min, 37 ^{o}C). Images of cells were recorded using a 12-bit grey-scale camera (Hammamatsu 8480) controlled by 'OpenLab' acquisition software (Improvision). Sequential images were acquired at excitation wavelengths of 350 and 380 nm and emitted light captured at 520 nm. Exposure times were normally within the range 250 to 2500 msec per frame. Post acquisition analysis was also undertaken using 'OpenLab' where 350 and 380 nm images were ratioed after background subtraction. In single cell high resolution experiments the 360/380 ratio images were subjected to a calibration procedure using the equation $[Ca^{2+]}_I = Kd^* (R-R_{min}) / (R_{max}-R)$. The parameters Kd* (1055), Rmin (.33) and Rmax (1.99) were determined in separate experiments using calibrated whole-cell intracellular solutions. $[Ca^{2+}]_I$ was estimated from within the cytoplasm defined regions of interest (ROI) of (>10 μm^2) and the mean calcium concentration within this area plotted as two dimensional graphs. Each concentration represents average responses of 20-30 cells from representative experiments. All experiments were repeated on more than three separate occasions. The maximum final concentration of DMSO (HC-030031, cinnamaldehyde, allyl isothiocyanate) and ethanol (Menthol and 4-hydroxy-2-nonenal) was 0.1%. Stock solutions were stored at -20˚C (with the exception of 4-hydroxy-2-nonenal which was stored at -80˚C) and diluted in extracellular solution immediately before use.

Reference List

Abrahamsen,B., Zhao,J., Asante,C.O., Cendan,C.M., Marsh,S., Martinez-Barbera,J.P., Nassar,M.A., Dickenson,A.H., and Wood,J.N. (2008). The cell and molecular basis of mechanical, cold, and inflammatory pain. Science *321*, 702-705.

Anand,U., Otto,W.R., Facer,P., Zebda,N., Selmer,I., Gunthorpe,M.J., Chessell,I.P., Sinisi,M., Birch,R., and Anand,P. (2008). TRPA1 receptor localisation in the human peripheral nervous system and functional studies in cultured human and rat sensory neurons. Neurosci.Lett. *438*, 221-227.

Auer-Grumbach,M., Olschewski,A., Papic,L., Kremer,H., McEntagart,M.E., Uhrig,S., Fischer,C., Frohlich,E., Balint,Z., Tang,B., et al. (2010). Alterations in the ankyrin domain of TRPV4 cause congenital distal SMA, scapuloperoneal SMA and HMSN2C. Nat.Genet. *42*, 160-164.

Bandell,M., Story,G.M., Hwang,S.W., Viswanath,V., Eid,S.R., Petrus,M.J., Earley,T.J., and Patapoutian,A. (2004). Noxious cold ion channel TRPA1 is activated by pungent compounds and bradykinin. Neuron *41*, 849-857.

Bautista,D.M., Jordt,S.E., Nikai,T., Tsuruda,P.R., Read,A.J., Poblete,J., Yamoah,E.N., Basbaum,A.I., and Julius,D. (2006). TRPA1 mediates the inflammatory actions of environmental irritants and proalgesic agents. Cell *124*, 1269-1282.

Bedoya,G., Montoya,P., Garcia,J., Soto,I., Bourgeois,S., Carvajal,L., Labuda,D., Alvarez,V., Ospina,J., Hedrick,P.W., et al. (2006). Admixture dynamics in Hispanics: a shift in the nuclear genetic ancestry of a South American population isolate. Proc.Natl.Acad.Sci.U.S.A *103*, 7234-7239.

Chen,J., Zhang,X.F., Kort,M.E., Huth,J.R., Sun,C., Miesbauer,L.J., Cassar,S.C., Neelands,T., Scott,V.E., Moreland,R.B., et al. (2008). Molecular determinants of species-specific activation or blockade of TRPA1 channels. J.Neurosci. *28*, 5063-5071.

Cox,J.J., Reimann,F., Nicholas,A.K., Thornton,G., Roberts,E., Springell,K., Karbani,G., Jafri,H., Mannan,J., Raashid,Y., et al. (2006). An SCN9A channelopathy causes congenital inability to experience pain. Nature *444*, 894-898.

Cregg,R., Momin,A., Rugiero,F., Wood,J.N., and Zhao,J. (2010). Pain Channelopathies. J.Physiol.

Deng,H.X., Klein,C.J., Yan,J., Shi,Y., Wu,Y., Fecto,F., Yau,H.J., Yang,Y., Zhai,H., Siddique,N., et al. (2010). Scapuloperoneal spinal muscular atrophy and CMT2C are allelic disorders caused by alterations in TRPV4. Nat.Genet. *42*, 165-169.

Dib-Hajj,S.D., Cummins,T.R., Black,J.A., and Waxman,S.G. (2007). From genes to pain: Na v 1.7 and human pain disorders. Trends Neurosci. *30*, 555-563.

Drenth,J.P. and Waxman,S.G. (2007). Mutations in sodium-channel gene SCN9A cause a spectrum of human genetic pain disorders. J.Clin.Invest *117*, 3603-3609.

Eid,S.R., Crown,E.D., Moore,E.L., Liang,H.A., Choong,K.C., Dima,S., Henze,D.A., Kane,S.A., and Urban,M.O. (2008). HC-030031, a TRPA1 selective antagonist, attenuates inflammatory- and neuropathy-induced mechanical hypersensitivity. Mol.Pain *4*, 48.

Einarsdottir,E., Carlsson,A., Minde,J., Toolanen,G., Svensson,O., Solders,G., Holmgren,G., Holmberg,D., and Holmberg,M. (2004). A mutation in the nerve growth factor beta gene (NGFB) causes loss of pain perception. Hum.Mol.Genet. *13*, 799-805.

Fertleman,C.R., Baker,M.D., Parker,K.A., Moffatt,S., Elmslie,F.V., Abrahamsen,B., Ostman,J., Klugbauer,N., Wood,J.N., Gardiner,R.M., et al. (2006). SCN9A mutations in paroxysmal extreme pain disorder: allelic variants underlie distinct channel defects and phenotypes. Neuron *52*, 767-774.

Foulkes,T. and Wood,J.N. (2008). Pain Genes. Plos Genetics *4*.

Hinman,A., Chuang,H.H., Bautista,D.M., and Julius,D. (2006). TRP channel activation by reversible covalent modification. Proc.Natl.Acad.Sci.U.S.A *103*, 19564-19568.

Indo,Y. (2001). Molecular basis of congenital insensitivity to pain with anhidrosis (CIPA): mutations and polymorphisms in TRKA (NTRK1) gene encoding the receptor tyrosine kinase for nerve growth factor. Hum.Mutat. *18*, 462-471.

Jordt,S.E., Bautista,D.M., Chuang,H.H., McKemy,D.D., Zygmunt,P.M., Hogestatt,E.D., Meng,I.D., and Julius,D. (2004). Mustard oils and cannabinoids excite sensory nerve fibres through the TRP channel ANKTM1. Nature *427*, 260-265.

Karashima,Y., Talavera,K., Everaerts,W., Janssens,A., Kwan,K.Y., Vennekens,R., Nilius,B., and Voets,T. (2009). TRPA1 acts as a cold sensor in vitro and in vivo. Proc.Natl.Acad.Sci.U.S.A *106*, 1273-1278.

Kim,H., Mittal,D.P., Iadarola,M.J., and Dionne,R.A. (2006). Genetic predictors for acute experimental cold and heat pain sensitivity in humans. J.Med.Genet. *43*, e40.

Koltzenburg,M., Lundberg,L.E., and Torebjork,H.E. (1992). Dynamic and static components of mechanical hyperalgesia in human hairy skin. Pain *51*, 207-219.

Krakow,D., Vriens,J., Camacho,N., Luong,P., Deixler,H., Funari,T.L., Bacino,C.A., Irons,M.B., Holm,I.A., Sadler,L., et al. (2009). Mutations in the gene encoding the calcium-permeable ion channel TRPV4 produce spondylometaphyseal dysplasia, Kozlowski type and metatropic dysplasia. Am.J.Hum.Genet. *84*, 307-315.

Kullmann,D.M. and Hanna,M.G. (2002). Neurological disorders caused by inherited ion-channel mutations. Lancet Neurol. *1*, 157-166.

Kwan,K.Y., Allchorne,A.J., Vollrath,M.A., Christensen,A.P., Zhang,D.S., Woolf,C.J., and Corey,D.P. (2006). TRPA1 contributes to cold, mechanical, and chemical nociception but is not essential for hair-cell transduction. Neuron *50*, 277-289.

Kwan,K.Y., Glazer,J.M., Corey,D.P., Rice,F.L., and Stucky,C.L. (2009). TRPA1 modulates mechanotransduction in cutaneous sensory neurons. J.Neurosci. *29*, 4808-4819.

Landoure,G., Zdebik,A.A., Martinez,T.L., Burnett,B.G., Stanescu,H.C., Inada,H., Shi,Y., Taye,A.A., Kong,L., Munns,C.H., et al. (2010). Mutations in TRPV4 cause Charcot-Marie-Tooth disease type 2C. Nat.Genet. *42*, 170-174.

Lathrop,G.M. and Lalouel,J.M. (1984). Easy calculations of lod scores and genetic risks on small computers. Am.J.Hum.Genet. *36*, 460-465.

Lathrop,G.M., Lalouel,J.M., Julier,C., and Ott,J. (1984). Strategies for multilocus linkage analysis in humans. Proc.Natl.Acad.Sci.U.S.A *81*, 3443-3446.

Lathrop,G.M., Lalouel,J.M., and White,R.L. (1986). Construction of human linkage maps: likelihood calculations for multilocus linkage analysis. Genet.Epidemiol. *3*, 39-52.

Lauria,G., Lombardi,R., Borgna,M., Penza,P., Bianchi,R., Savino,C., Canta,A., Nicolini,G., Marmiroli,P., and Cavaletti,G. (2005). Intraepidermal nerve fiber density in rat foot pad: neuropathologic-neurophysiologic correlation. J.Peripher.Nerv.Syst. *10*, 202-208.

Lauria,G., Cornblath,D.R., Johansson,O., McArthur,J.C., Mellgren,S.I., Nolano,M., Rosenberg,N., and Sommer,C. (2005). EFNS guidelines on the use of skin biopsy in the diagnosis of peripheral neuropathy. Eur.J.Neurol. *12*, 747-758.

Macpherson,L.J., Dubin,A.E., Evans,M.J., Marr,F., Schultz,P.G., Cravatt,B.F., and Patapoutian,A. (2007). Noxious compounds activate TRPA1 ion channels through covalent modification of cysteines. Nature *445*, 541-545.

Martikainen,I.K., Narhi,M.V., and Pertovaara,A. (2004). Spatial integration of cold pressor pain sensation in humans. Neurosci.Lett. *361*, 140-143.

Materazzi,S., Nassini,R., Andre,E., Campi,B., Amadesi,S., Trevisani,M., Bunnett,N.W., Patacchini,R., and Geppetti,P. (2008). Cox-dependent fatty acid metabolites cause pain through activation of the irritant receptor TRPA1. Proc.Natl.Acad.Sci.U.S.A *105*, 12045-12050.

McMahon,S.B. and Wood,J.N. (2006). Increasingly irritable and close to tears: TRPA1 in inflammatory pain. Cell *124*, 1123-1125.

Munns,C., AlQatari,M., and Koltzenburg,M. (2007). Many cold sensitive peripheral neurons of the mouse do not express TRPM8 or TRPA1. Cell Calcium *41*, 331-342.

Nagatomo,K. and Kubo,Y. (2008). Caffeine activates mouse TRPA1 channels but suppresses human TRPA1 channels. Proc.Natl.Acad.Sci.U.S.A *105*, 17373-17378.

Nilius,B. (2007). TRP channels in disease. Biochim.Biophys.Acta *1772*, 805-812.

Norbury,T.A., MacGregor,A.J., Urwin,J., Spector,T.D., and McMahon,S.B. (2007). Heritability of responses to painful stimuli in women: a classical twin study. Brain *130*, 3041-3049.

Peterlin,Z., Chesler,A., and Firestein,S. (2007). A painful trp can be a bonding experience. Neuron *53*, 635-638.

Prober,D.A., Zimmerman,S., Myers,B.R., McDermott,B.M., Jr., Kim,S.H., Caron,S., Rihel,J., Solnica-Krezel,L., Julius,D., Hudspeth,A.J., et al. (2008). Zebrafish TRPA1 channels are required for chemosensation but not for thermosensation or mechanosensory hair cell function. J.Neurosci. *28*, 10102-10110.

Rock,M.J., Prenen,J., Funari,V.A., Funari,T.L., Merriman,B., Nelson,S.F., Lachman,R.S., Wilcox,W.R., Reyno,S., Quadrelli,R., et al. (2008). Gain-of-function mutations in TRPV4 cause autosomal dominant brachyolmia. Nat.Genet. *40*, 999-1003.

Rugiero,F. and Wood,J.N. (2009). The mechanosensitive cell line ND-C does not express functional thermoTRP channels. Neuropharmacology *56*, 1138-1146.

Sobel,E. and Lange,K. (1996). Descent graphs in pedigree analysis: applications to haplotyping, location scores, and marker-sharing statistics. Am.J.Hum.Genet. *58*, 1323-1337.

Story,G.M., Peier,A.M., Reeve,A.J., Eid,S.R., Mosbacher,J., Hricik,T.R., Earley,T.J., Hergarden,A.C., Andersson,D.A., Hwang,S.W., et al. (2003). ANKTM1, a TRP-like channel expressed in nociceptive neurons, is activated by cold temperatures. Cell *112*, 819-829.

Taylor-Clark,T.E., Ghatta,S., Bettner,W., and Undem,B.J. (2009). Nitrooleic acid, an endogenous product of nitrative stress, activates nociceptive sensory nerves via the direct activation of TRPA1. Mol.Pharmacol. *75*, 820-829.

Trevisani,M., Siemens,J., Materazzi,S., Bautista,D.M., Nassini,R., Campi,B., Imamachi,N., Andre,E., Patacchini,R., Cottrell,G.S., et al. (2007). 4-Hydroxynonenal, an endogenous aldehyde, causes pain and neurogenic inflammation through activation of the irritant receptor TRPA1. Proc.Natl.Acad.Sci.U.S.A *104*, 13519-13524.

Velasco,M., Garcia,E., and Onetti,C.G. (2006). Glucose deprivation activates diversity of potassium channels in cultured rat hippocampal neurons. Cell Mol.Neurobiol. *26*, 307-319.

Voets,T., Droogmans,G., Wissenbach,U., Janssens,A., Flockerzi,V., and Nilius,B. (2004). The principle of temperature-dependent gating in cold- and heat-sensitive TRP channels. Nature *430*, 748-754.

Voets,T., Owsianik,G., Janssens,A., Talavera,K., and Nilius,B. (2007). TRPM8 voltage sensor mutants reveal a mechanism for integrating thermal and chemical stimuli. Nat.Chem.Biol. *3*, 174-182.

Wang,A.K., Fealey,R.D., Gehrking,T.L., and Low,P.A. (2008). Patterns of neuropathy and autonomic failure in patients with amyloidosis. Mayo Clin.Proc. *83*, 1226-1230.

Xiao,B., Dubin,A.E., Bursulaya,B., Viswanath,V., Jegla,T.J., and Patapoutian,A. (2008). Identification of transmembrane domain 5 as a critical molecular determinant of menthol sensitivity in mammalian TRPA1 channels. J.Neurosci. *28*, 9640-9651.

Yang,Y., Wang,Y., Li,S., Xu,Z., Li,H., Ma,L., Fan,J., Bu,D., Liu,B., Fan,Z., et al. (2004). Mutations in SCN9A, encoding a sodium channel alpha subunit, in patients with primary erythermalgia. J.Med.Genet. *41*, 171-174.

Zarate,Y.A. and Hopkin,R.J. (2008). Fabry's disease. Lancet *372*, 1427-1435.

Zhang,Y. and Lipton,P. (1999). Cytosolic Ca2+ changes during in vitro ischemia in rat hippocampal slices: major roles for glutamate and Na+-dependent Ca2+ release from mitochondria. J.Neurosci. *19*, 3307-3315.

Zurborg,S., Yurgionas,B., Jira,J.A., Caspani,O., and Heppenstall,P.A. (2007). Direct activation of the ion channel TRPA1 by Ca2+. Nat.Neurosci. *10*, 277-279.
